# Psychometric properties of the mental health continuum-short form in Iranian adolescents

**DOI:** 10.3389/fpsyg.2023.1096218

**Published:** 2023-02-09

**Authors:** Majid Yousefi Afrashteh, Parisa Janjani

**Affiliations:** ^1^Department of Psychology, Faculty of Humanities, University of Zanjan, Zanjan, Iran; ^2^Cardiovascular Research Center, Imam Ali Hospital, Health Institute, Kermanshah University of Medical Sciences, Kermanshah, Kerman, Iran

**Keywords:** mental health, mental health continuum-short form, psychometric, adolescents, Iran

## Abstract

**Introduction:**

Psychological tests are necessary to assess the mental state of individuals. Mental health is one of the important psychological indicators and is increasingly considered as having various aspects of well-being. The Mental Health Continuum-Short Form (MHC-SF) is a 14-item instrument that assesses mental health, focusing on emotional, psychological, and social well-being. The present study, the psychometric properties of the Persian version of the MHC-SF were examined in relation to its factor structure, internal consistency, construct validity, and gender measurement invariance among adolescents.

**Methods:**

The population of this study was Iranian adolescents between 11-and 18-year-old who were enrolled in the seventh to twelfth grades. A convenience sample of 822 Adolescents from four large cities in the Iran (Tehran, Zanjan, Hamedan and Ghazvin) participated in the present study. Questionnaires were completed online. Statistical analyses to evaluate the factor structure, internal consistency, construct validity, gender and age factorial invariance were performed in SPSS and LISREL.

**Results:**

According to the results of confirmatory factor analysis, the MHC-SF is composed of three factors: emotional, psychological, and social well-being. Reliability was confirmed by Cronbach’s alpha method and composite reliability (>0.7). Measurement invariance were confirmed among girls and boys. Convergent and divergent validity were also evaluated and confirmed by correlating the test score with similar and different tests.

**Conclusion:**

This study confirmed the psychometric properties of MHC-SF in the Iranian adolescent community. This instrument can be used in psychological research and diagnostic evaluations.

## Introduction

Adolescence is a critical period for increasing vulnerability and the onset of mental disorder (World Health Organization, 2014; Heizomi et al., 2020). Mental health problems during adolescence impose psychological, social and economic challenges on any society (Kieling et al., 2011; Sharifi et al., 2016; Heizomi et al., 2020). Epidemiological studies have shown the prevalence of adolescent mental disorder from 10 to 20% worldwide (Kieling et al., 2011). About 15% of Iran’s population is between 10 and 20 years old and adolescents (Statistical Center of Iran, 2016). Based on epistemological data in Iran, behavioral and mental health problems are common in this group (Mohammadi et al., 2014; Sharifi et al., 2015; Rogoza et al., 2018). In national epidemiological survey in of Iranian Children and adolescents, 6,209 out of 30,532 (22.31%) were diagnosed with at least one psychiatric disorder. The anxiety disorders (14.13%) and behavioral disorders (8.3%) had the highest prevalence, while eating disorders (0.13%) and psychotic symptoms (0.26%) had the lowest. In other words, about one fifth of Iranian children and adolescents suffer from at least one psychiatric disorder (Mohammadi et al., 2019). Regarding anxiety and depression, emotion control skills develop considerably over adolescence. Adolescence is additionally a threat period for the modern onset of anxiety and depressive disorders, psychopathologies which have long been related to disturbances in the control of positive and negative emotion (Young et al., 2019). Emotion control is characterized as the capacity to oversee one’s passionate reactions. This incorporates techniques to extend, keep up, or decrease the intensity, length, and direction of positive and negative emotion (Parrott, 1993; Gross, 2002; Koole, 2010). Learning to direct emotion may be a critical socio-emotional ability that permits adaptability in emotionally-evocative circumstances. There are precise formative shifts in how we oversee enthusiastic reactions. In early childhood, feelings are communicated and external support is sought (Kopp, 1989). In adolescence, there is ordinarily a diminished dependence on parental support and constrained adequacy of adaptive inside emotion control (Zimmermann and Iwanski, 2014). Disturbances to emotion control capacities in adulthood are central to hypotheses of how will manifest anxiety and depressive disorders and also be maintained (Hofmann et al., 2012). These hypotheses recommend that decreased capacities to downregulate increased negative influence are typical to both anxiety and depression, though the diminished ability to direct positive affect may be more particular to depressive disorders (Werner-Seidler et al., 2013). Adolescence may be a period of increased hazard for the onset of anxiety-clutters and depression (Beesdo et al., 2010; Lee et al., 2014). It is well-established that stressful life occasions and childhood difficulties are significant risk factors for future psychopathology (Kessler et al., 2010).

The relationship between mental health and mental disorder has evolved in recent decades, and this has influenced the conceptualization of mental health. For example, the World Health Organization defined mental health as being free from mental illness (Snyder et al., 2002; Fen et al., 2013). However, focusing solely on the prevention and treatment of mental disorder was not successful in reducing the prevalence of mental disorder (Insel and Scolnick, 2006). In particular, Weisz et al. (2017) showed that in the last 50 years, psychological interventions in children and adolescents have not led to much improvement. On the other hand, the pathological view of mental health did not help to distinguish adolescents with educational and behavioral problems (Antaramian et al., 2010).

With the development of positive psychology, a new approach to mental health was formed. In the latest definition of the World Health Organization (World Health Organization, 2004) mental health is defined as “A state of well-being in which every individual realizes his or her own potential, can cope with the normal stresses of life, can work productively and fruitfully, and is able to make a contribution to her or his community.” These approaches emphasize the role of positive functioning, worthwhile goals, meaningful activity and optimistic growth in mental well-being. Thus, elements of positive mental health and symptoms of mental disorder can coexist. In this view, the strengths and weaknesses of the individual are seen together and must be combined to fully assess the mental state.

Various models have been proposed for conceptualizing and evaluating mental health. In Keyes’s theory (Keyes, 2002), human beings are embedded in social structures, face various social challenges, and have numerous interpersonal interactions, thus addressing the social aspect of mental health. Keyes (2002, 2007) developed two distinct but related continuum model, instead of a single continuum with mental well-being and illness at both ends. This mental health continuum distinguishes three levels of positive mental health: flourishing, moderate, and languishing mental health (Keyes et al., 2012). In the theoretical model of MHC, it is separated into three factors “emotional, psychological and social.” Emotional well-being includes of positive emotions and satisfaction life (Diener et al., 1999). Psychological well-being comprises aspects of individuals’ psychological functioning (e.g., autonomy and a sense of personal growth; Ryff, 1989). Social well-being focuses on individuals’ evaluations of their social lives, capturing individuals’ appraisals of their own circumstances and functioning in society (Keyes, 1998, 2002). Keyes (2002) has argued that it takes a blend of emotional, psychological, and social well-being to be considered mentally healthy. He distinguishes flourishing as a state where individuals combine a high level of subjective well-being with an optimal level of psychological and social functioning. Similarly, languishing refers to a state in which low levels of subjective well-being are combined with low levels of psychological and social well-being. Those who are not declining and flourishing have average mental health. This definition of low levels of subjective well-being parallels the definition of depression in the DSM-IV, which includes both feelings of anhedonia (feeling sad or loss of interest and pleasure) and reported problems in functioning (such as problems in appetite, sleeping, or fatigue) (Keyes, 2002). People with flourishing mental health have enjoyable and positive performance (Keyes, 2005). In contrast, people with languishing mental health experience low pleasure. Epidemiological studies have shown that MHC-SF is associated with superior physical, mental, and psychosocial functions.

Most instruments designed to measure adolescent mental well-being either have many questions or measure limited dimensions of mental health (Luijten et al., 2019). Acceptance of MHC-SF psychometric properties in many countries supports its desirability, validity and reliability. The 14 items continuum-short form of mentalhealth (MHC-SF) (Singh et al., 2015) is a short questionnaire that corresponds to the 40 item Continuum mental health form (Donnelly et al., 2019). Psychometric properties of the MHC-SF were confirmed in adolescents and adults of different cultures, including Argentina (Perugini et al., 2017), Canada (Doré et al., 2017), China (Guo et al., 2015), Egypt (Salama-Younes, 2011), India (Singh et al., 2015), Ireland (Donnelly et al., 2019), Italy (Petrillo et al., 2015), Korea (Lim, 2014), Poland (Karaś et al., 2014), South Africa (Keyes et al., 2008), United States (Keyes, 2006b; Keyes et al., 2012), Dutch (Luijten et al., 2019). In Iran, too, Joshanloo (2016) reported the psychometric properties of this tool well. But his research sample was university students.

As mentioned, adolescents are a special group and the structure obtained from adults about them may not be valid. Given that the field of planning and exercise is increasingly focused on mental health among adolescents, an appropriate instrument for this age group is needed to considering the variance in well-being. The aim of this study was to adapt MHC-SF to a sample of adolescents in Iranian society. For this purpose, the factor structure and psychometric properties have been evaluated. Most previous research has confirmed the three-factor structure for MHC-SF (Guo et al., 2015; Doré et al., 2017; Perugini et al., 2017). In order to investigate the psychometric properties of MHC-SF, first, its reliability is evaluated based on internal consistency and composite reliability, second, determining the factor structure by assuming three underlying factors, and third, evaluating construct validity of the MHC-SF-A, correlating the PANAS-C, and Kidscreen-2 and DASS-21.

## Material and method

### Participants

The population of this study was Iranian adolescents between 11 and 18 years old who were enrolled in the seventh to twelfth grades. A convenience sample of 822 adolescents from four large cities in the Iran (Tehran, Zanjan, Hamedan and Ghazvin) participated in the present study. They were relatively proportional distributed by sex: 430 girls (52%) and 392 boys (48%). The mean age was 16.33 years old (*SD* = 8.80). The highest percentage of the participants (38.6%; *n* = 317) lived in Tehran province, 20% (*n* = 165) lived in Zanjan province, 22% (*n* = 179) lived in Hamedan province 18.7% (*n* = 154) lived in Ghazvin province and only 7 cases did not report their residence. Concerning their socioeconomic status, the majority (68.1%, *n* = 560) described itself as belonging to the middle class, 19.9% (*n* = 164) to middle-low or lower class, 9.2% (*n* = 76) to high or middle-high class, and 2.6% (*n* = 22) did not report their class. In the Iranian educational system, the first and second secondary education are included from the seventh to the 12 grades. Most of the participants (58%, n = 479) were enrolled in the second secondary, compared to 42% (n = 345) who were enrolled in the first secondary education. In terms of educational grade, the sample consisted of 95 7th graders (11.5%), 115 8th graders (13.9%), 135 9th graders (36.5%), 164 10th graders (36.5%), 195 11th graders (36.5%), and 120 12th graders (36.5%).

### Procedure

The executive process of this research has been approved by the Ethics Committee of Kermanshah University of Medical Sciences under No. IR.KUMS.REC.1400.608 all procedures were carried out an adequate understanding and each participant provided their informed consent prior to the study. Data were collected through non-random and voluntary sampling. Iranian adolescents were asked to complete online questionnaires. Questionnaires were provided for online implementation and administered from November 28th 2020 to February 16th 2021. Before completing the questionnaires, the participants were explained the purposes and significance of research and their informed consent was obtained. For subjects under 16 years of age, the questionnaire link was first provided to their parents and after their consent, the questionnaire link was provided to their children.

### MHC-sf-a

The original 14-item Mental Health Continuum–Short Form (MHC-SF) (Keyes et al., 2008) is a self-report questionnaire, measuring three basic subjective well-being domains: emotional (3 items), psychological (6 items) and social (5 items) of well-being. Respondents rated the frequency of every feeling in the past month on a 6-point Likert scale. Respondents thought about their past month and rated the frequency of each feeling on a 6-point Likert-type scale, from never (0) to every day (5). The Iranian version of this questionnaire has already been used and validate by Rafiey et al. (2017) in the adult population. The original English MHC-SF for adolescent is just like the adult version, with only one helpful change to better fit the adolescent population (Keyes et al., 2008). Specially, examples of the community in the item “How often did you feel that you belonged to a community?” which in the adult version was “(like a social group, your neighborhood, or your city)” were given in the adolescent version as “(like a group of friends, at school, or in the neighborhood).”

### PANAS-C

The positive affect (PA) dimension of the 10-item PANAS-C (Ebesutani et al., 2012) was selected to evaluate emotional well-being, as referred to the degree to which people feel are vitality and enthusiastic. The PA dimension was evaluated five adjective by five items: happy, lively, happy, energetic, and proud. The items have a 5-point Likert response format, with answers ranging from 1 („very little) to 5 („a lot’). The sum of the item scores gives the total health score. The PA dimension has been shown to measure PA markers well among 6–18-year-olds. Ebesutani et al. (2012) showed that PANAS-C is valid and reliable for the age group of 18–18 years. Lotfi et al. (2020) reported the psychometric properties of this questionnaire very well in Iran. In the present study, the Cronbach’s alpha of the PANAS-C was 0.76.

### Kidscreen-27

Kidscreen-27 (Ravens-Sieberer et al., 2008) is a brief screening measure to evaluate the behavioral and emotional problems of children and adolescents by 27 items measuring five scales, physical well-being, psychological well-being, autonomy and parents, peers and social support, and school environment. Items are scored on a 5-point Likert scale. The higher the total score indicates greater quality of life. Nik-Azin et al. (2013) reported the psychometric properties of this questionnaire in Iran, suitable for the age group of 11 to 19 years. The results of this study supported the five-factor structure of the original version. In the present study, the Cronbach’s alpha of the Kidscreen-27 was 0.73.

### DASS-21

The DASS-21 (Antony et al., 1998) is a short form of DASS-42, well-established instrument for measuring depression, anxiety, and stress with good reliability and validity reported in different cultural context (Oei et al., 2013). DASS-21 is a set of three self-report 7-item scales for assessing negative mental states in anxiety, depression, and stress. All 21 items are scored on a 4-point Likert scale from 0) did not apply to me at all) to 3 (applied to me very much, or most of the time). Asghari et al. (2008), examining the psychometric properties of this questionnaire in Iran, reported it as valid and reliable. A high score indicates psychological distress on each scale. In the present study, the Cronbach’s alpha of the DASS-21 was 0.75. In the section, only the “anxiety” and “depression” dimensions of that scale were used for the purposes of this study.

### Data analysis method

After data collection and data screening in the first stage, and after discarding 21 questionnaires with missing or distorted data, the main analyzes were performed with SPSS-26 and Lisrel-10.2 software.

#### Face validity

The purpose of face validity is to ensure that respondents understand the items. In this study, face validity was evaluated quantitatively and qualitatively. For qualitative face validity, the questionnaire was provided to 15 participants of the target population to determine the degree of appropriateness, the level of difficulty, and the ambiguity of the items. For quantitative face validity, 30 adolescents determined the importance of the items in relation to the goal of the study. The impact score of each item was measured based on the formula: average × ratio of individuals who have chosen the most important and important option divided by the total number of individuals. The items with an impact score of more than 1.5 were accepted (Asghari et al., 2008).

#### Content validity

Content validity was evaluated both quantitatively and qualitatively. To evaluate the quantitative content validity, an expert panel of 10 people was formed including 8 children and adolescent psychologists and 2 psychometricians and they were asked to comment on the necessity of each item. Based on this, the value of content validity index (CVI) and Content Validity Ratio (CVR) was calculated. As a criterion, the acceptable value for CVI of each item is 0.7 and more. The expert panel was also asked to rate the items of the questionnaire in terms of clarity of the items. To test qualitative content validity, the experts provided their comments on grammar, editing points, use of appropriate words, sentence structure, etc. for each ite.

#### Factorial validity

After confirming the face validity and content of the items, the factorial validity was assessed using confirmatory factor analysis. LISREL10.2 was used to evaluate the factor structure. The method of estimating the weighted least squares with data from polychoric matrix and asymptotic covariance matrix was used in data analysis. The least squares method was preferred because the Likert response options were five-choice and the polychoric matrix had to be calculated instead of the Pearson correlation (Keyes, 1998).

In this phase, 801 adolescents participated. The model was evaluated using fit indices of chi-square, chi-square to the degree of freedom ratio (*X*^2^/df), standard deviation estimation error (Root Mean Square Error of Approximation), goodness of fit index (GFI), adjusted goodness of fit index (AGFI), Parsimony goodness of fit index (PGFI), Normed Fit Index (NFI) and comparative fit index (CFI) were used. *p*-value more than.05, *X*^2^/df less than three and RMSEA more than 0.08, PGFI more than 0.5, and other indices more than 0.9 were accepted (Schreiber et al., 2006).

#### Measurement invariance

Multigroup confirmatory factor analysis was performed to evaluate the invariance of the best-fitting model based on gender. Four types of invariance were investigated in this study: configural invariance (Is the configuration of the model the same across groups?), metric/weak invariance (Are factor loadings the same across groups?), scalar/strong invariance (Are the intercepts the same across groups?), and strict invariance (Are the residual variances the same across groups?) across gender (boys vs. girls). Configural invariance was confirmed if RSMEA and SRMR were < 0.08 and CFA was >0.95 (Cheung and Rensvold, 2002). A relative change of ≤0.010 in CFI, supplemented by a relative change of ≤0.015 in RMSEA or ≤ 0.030 in SRMR, indicated that the null hypothesis of invariance should not be rejected (Chen, 2007).

#### Construct validity

To evaluate the construct validity, the relationship between the score obtained from MHC-SF-A and several other measures was examined. According to Keyes (2007) conceptualization, MHC-SF-A is expected to be negatively related to anxiety and depression (convergent validity) and also has a positive relationship with Kidscreen-27 positive affect tests (divergent validity). The Pearson correlation coefficient was used to determine the direction and intensity of the relationship between the measures.

#### Reliability

The reliability of the MHC-SF was determined through internal consistency and composite reliability. Value greater than 0.7 was considered acceptable (Keyes, 2006a).

## Results

The results of the analysis are reported separately for different areas of psychometrics.

Table 1 shows the descriptive statistics (means, standard deviations, skewness, and kurtosis), Cronbach alphas and composite reliabilities for latent MHC-SF-A subscales.

**Table 1:** Descriptive statistics for MHC-SF dimensions.

Dimension	M	SD	Skewness	Kurtosis
Emotional	10.31	3.17	0.38	0.70
Social	14.82	3.94	0.74	0.67
Psychological	18.13	4.26	0.43	0.69
MHC-SF Total	43.27	12.48	0.51	0.33

### Face validity

In order to achieve qualitative face validity, the opinion of the experts of the specialized panel was applied. Given the acceptable impact score value, the quantitative face validity of items was also confirmed. However, “That you had something important to contribute to society” received the maximum score (IS = 4.1), whereas, the minimum score was attributed to “That you liked most parts of your personality” (Is = 2.16).

### Content validity

The CVR and CVI of the questionnaire were evaluated according to the opinions of the expert’s panel. The values of both indicators for all questions were between 0.8 and 0.98. Therefore, the content validity of the MHC-SF was confirmed.

### Factorial validity

Confirmatory factor analysis was performed by comparing four models of one-factor, two-factor, three-factor first-order and three-factor second-order. According to the information in Table 2, the first-order three-factor model has the best fit. Table 3 reports the standard estimates and the t-value based on the best fit of the factor model.

**Table 2:** Results of the confirmatory factor analyses.

Model	*X* ^2^	df	IFI	CFI	NFI	RMSEA	90% CI RMSEA
Single factor	727.22	77	0.823	0.818	0.813	0.131	0.126–0.136
Two factor	498.09	75	0.872	0.868	0.867	0.111	0.106–0.116
Three factor	222.35	73	0.938	0.943	0.941	0.061	0.055–0.066
Second order (three factor)	315.74	68	0.910	0.901	0.903	0.082	0.074–0.087

*χ^2^*: Chi Squared test, df: degrees of freedom, IFI: Incremental Fit Index, CFI: comparative fit index, NFI: Normed-of-Fit Index, RMSEA: root-mean-square error of approximation, CI: confidence interval. Criteria for interpreting model fit are: RSMEA < 0.08, IFI, CFI and NFI > 0.90.

**Table 3:** Standard estimate and *t*-value for the relationship between the item and the factor in the three-factor model.

Factor	Item	Factor loading	*t*-value
Emotional Wellbeing	Happy	0.65	7.31
	Interested in life	0.58	6.25
	Satisfied	0.55	6.04
Social Wellbeing	That you had something important to contribute to society	0.61	7.09
	That you belonged to a community (like a group of friends, at school or in the neighborhood)	0.51	5.87
	That our society is becoming a better place for people	0.57	6.16
	That people are basically good	0.58	6.20
	That the way our society works makes sense to you	0.59	6.29
Psychological Wellbeing	That you liked most parts of your personality	0.63	7.24
	Good at managing the responsibilities of your daily life	0.57	6.18
	That you had warm and trusting relationships with others	0.60	6.34
	That you have experiences that challenge you to grow and become a better person	0.64	7.28
	Confident to think or express your own ideas and opinions	0.49	5.53
	That your life has a sense of direction or meaning to it	0.63	7.20

### Measurement invariance

Table 4 shows the values for comparing models and measurement variability.

**Table 4:** Measurement invariance across gender.

model	*X* ^2^	df	CFI	RMSEA	Δ*X*^2^	Δdf	*p*	ΔCFI	ΔRMSEA
Configural invariance	339.33	149	0.942	0.062	–	–		–	–
Metric Invariance	352.85	160	0.940	0.061	13.52	11	0.260	0.002	0.001
Scalar invariance	362.19	164	0.942	0.060	22.86	15	0.087	0.000	0.002
Strict Invariance	379.43	178	0.938	0.061	40.10	29	0.082	0.004	0.001

According to the appropriate fit of the three-factor model between girls and boys, the configural invariance is confirmed. Changes of *X*^2^, CFI, and RMSEA, when the metric/weak invariance model is compared with the configural invariance model, were within recommended values (ΔCFI = 0.002, Δ*X*^2^ = 13.52 with *p* = 0.26, ΔRMSEA = 0.001). This indicates that the metric of factor scores was invariant across gender. This confirms the items used to estimate the factor loadings have the same meaning for males and females. The second more restrictive model, which constrained the factor loadings and item intercept to create the scalar/strong invariance model, resulted in the demonstration of strong invariance (ΔCFI = 0.000, Δ*X*^2^ = 22.86 with *p* = 0.087, ΔRMSEA = 0.002). This indicates that both factor loadings and item intercept are invariant between genders. The last more restrictive model, which constrained the factor loadings, item intercept, and residual variances, to produce the strict invariance model was then inspected. The changes of the fit indices were within the acceptable values (ΔCFI = 0.004, Δ*X*^2^ = 40.10 with *p* = 0.082, ΔRMSEA = 0.001).

### Construct validity

To evaluate the construct validity, the relationship between the score obtained from MHC-SF-A and several other measures was examined. According to Keyes (2007) conceptualization, MHC-SF-A is expected to be negatively related to anxiety and depression (divergent validity), also has a positive relationship with Kidscreen-27 positive affect tests (convergent validity). The results of Pearson correlation coefficient for convergent and divergent validity are reported in Table 5. This table also shows the relationship between MHC-SF-A subscales to evaluate the internal homogeneity of the test. Based on these results, there is both convergent validity, divergent validity and internal validity between MHC-SF-A subscales.

**Table 5:** Pearson correlation coefficients for construct validity.

Measure	MHC-SF subscale			MHC-SF total
	Emotional	Psychological	Social	
*Divergent validity*				
Anxiety (DASS-21)	−0.40***	−0.330***	−0.290***	−0.37***
Depression (DASS-21)	−0.430***	−0.370***	−0.360***	−0.40***
*Convergent validity*				
Quality of life (Kidscreen-27)	0.480***	0.470***	0.400***	0.45***
Positive affect (PANAS-C)	0.500***	0.43	0.380***	0.44***
*Internal Consistency*				
MHC-SF subscale				
Emotional	–			
Psychological	0.620***	–		
Social	0.500***	0.550***	–	

***p < 0.001.

### Reliability

The results reported in Table 6 support the reliability of the scales in both Cronbach’s alpha methods and the composite reliability (>0.70(that indicates that the MHC-SF is a reliable measure, therefore it can be accepted.

**Table 6:** Composite reliabilities and Cronbach alpha coefficients for MHC-SF-A subscales.

Scale	CR	*α*
Emotional	0.87	0.84
Psychological	0.85	0.85
Social	0.86	0.82
MHC-SF total	0.88	0.87

## Discussion

The aim of this study was to examine at the structure and psychometric features of data collected for teenagers using the Mental Health Continuum-Short Form version (Keyes et al., 2008) in Iranian adolescences, its internal consistency and reliability, its invariance across gender, and plausibility of the two continua model proposing that mental health and illness are distinct yet related constructs. The Mental Health Continuum, or MHC, provides a clinical approach toward the ongoing evaluation and categorical identification of positive mental health states (Keyes, 2007; Keyes et al., 2008). The 14-item short form of the MHC scale is one of the most extensively used measures to assess well-being around the world (Perugini et al., 2017). As a result, the primary goal of this research would have been to verify the MHC–SF in an Iranian adolescent population. The present results confirm that the MHC-SF is a valid and reliable tool that can be taken advantage of evaluating the health of Iranian adolescents.

The results of study prove that the multidimensional structure of well-being (emotional, social and psychological). The three-factor model showed an acceptable goodness-of-fit index and was relatively superior to the one-and two-factor models. These findings are consistent with a growing body of research suggesting that the MHCSF measures three specific factors that correspond to major components of overall well-being and subscales of emotional, social, and psychological well-being (Karaś et al., 2014; Petrillo et al., 2015; Joshanloo, 2016; Echeverría Errázuriz et al., 2017; Rogoza et al., 2018; Longo et al., 2020).

MHCSF emotional and psychometric scales in this population displayed high internal consistency and reliability, as assessed by Cronbach’s alpha. The internal consistency and reliability coefficients of the social well-being subscale were acceptable, but low compared to the other subscales. Similar results have been detected in preceding studies (Keyes et al., 2008; Lamers et al., 2011; Karaś et al., 2014; Petrillo et al., 2015). All Cronbach alphas were superior to those seen in studies in South Africa, the Netherlands and Italy (Keyes et al., 2008; Lamers et al., 2011; Karaś et al., 2014).

Confirmatory Factor Analysis (CFA) was calculated to determine the factor structure of MHC-SF. A second-order CFA was performed to test whether these three factors reveal the same dimension. The CFA provides a fairly good level of support for the MHC-SF tripartite structure (Joshanloo et al., 2013; Karaś et al., 2014). A unique cross-culture study of MHC-SF factor structure is Joshanloo et al. (2013) study using CFA, displaying that the three-dimensional model of the MHC-SF corresponded to the data well in Iran, South Africa, and the Netherlands. The overall Iranian MHC-SF and the three sub-dimensions were more internally consistent than other studies. In addition, our results confirmed strong invariance of the three MHC-SF elements by gender. These results suggest that MHC-SF is similarly measured in males and females using a three-factor model, allowing comparisons between genders.

The convergent validity of the MHCSF was good in the present study, implying that the MHC-SF is a valid instrument. The Pearson Correlation Coefficient results for convergent and divergent validity showed the relationship between the MHC-SF-A subscales for assessing the internal homogeneity of the test. Between anxiety and depression, depression covered a broader conceptualization of well-being. This means that depression on a health-affirming measure, for example, is most strongly correlated with the total score of the MHC-SF, a measure of general well-being. According to Keyes’ conceptualization (Keyes, 2002), MHC-SF-A is expected to be negatively related to anxiety and depression. Previous studies have also demonstrated the convergent validity of the emotional, social, and psychological aspects of mental well-being (Keyes et al., 2012; Joshanloo et al., 2013).

In addition to convergent validity, the MHC–SF was found to have divergent and discriminant validity in the current investigation. Mental disorder and mental health, according to the two-continua concept, are connected but separate dimensions. The current study demonstrates that mental health measures are connected to, but separate from, mental disorder measures. This obviously means that the lack of mental disorder does not always imply the existence of mental health, requiring the development of a mental health assessment tool.

Generally, the MHC-SF is a beneficial, short self-report questionnaire for evaluating of mental health. Consequently, research helps fill knowledge gaps about the authenticity and usefulness of the MHC-SF in national cultures around the world for positive mental health measures.

There are some limitations that need to be taken into account in this study. Weak correlations are statistically significant due to the large sample size. As a result, we applied an alpha of 0.001 instead of the common 0.05 as a margin of significance in the validation analyses. Due to the extremely limiting assumptions of the CFA approach and resulting inflation of the interrelationships, the future studies are capable of applying Exploratory Structural Equation Modeling (ESEM) to illustrate the factor structure of multidimensional constructs such as mental well-being. What is more, future research will be able to discover the validity of the Iranian MHC–SF, and the three categorizations for positive mental health and people in different sorting.

According to the results, there are some vital implications for mental health policy and care. Recently, mental health care concentrates mostly on psychopathology in either diagnostics or treatment. Nevertheless, with mental health and mental disorder being two detached indices of mental health, it may be useful to concentrate also on boosting of positive mental health. It is hoped that this study will cover the path for more knowledgeable and inclusive conceptualization and assessment of mental well-being in different age groups.

## Conclusion

In this research, in relation to the level of mental health, we administered the Mental Health Continuum-Short Form (Keyes et al., 2008) to a large Iranian sample (aged from 11 to 18 years) to examine its factor structure, psychometrics, and benefit in determining the level of functioning among Keyes’ (Keyes, 2002) model. Consequently, to our knowledge, the present study is among the first to confirm a measure of Iranian adolescences’ positive mental health and presents primary confirmation that the MHC-SF can be applied to younger participants. These results support the findings of prior research in terms of adolescents (Keyes, 2006a,b; Matos et al., 2010), implying that this conceptualization of mental health can be appropriate from immaturity to maturity. To sum up, we deliberate the MHC-SF to be a psychometrically sound questionnaire for general mental well-being in relation to Iranian adolescents.

## Data availability statement

The raw data supporting the conclusions of this article will be made available by the authors, without undue reservation.

## Ethics statement

The studies involving human participants were reviewed and approved by Ethics Committee of Kermanshah University of Medical Sciences. Written informed consent to participate in this study was provided by the participants’ legal guardian/next of kin.

## Author contributions

MYA conceived and designed the research; MYA and PJ collected, organized and analyzed the data; MYA wrote the paper. MYA and PJ read and approved the final manuscript.

## Conflict of interest

The authors declare that the research was conducted in the absence of any commercial or financial relationships that could be construed as a potential conflict of interest.

## Publisher’s note

All claims expressed in this article are solely those of the authors and do not necessarily represent those of their affiliated organizations, or those of the publisher, the editors and the reviewers. Any product that may be evaluated in this article, or claim that may be made by its manufacturer, is not guaranteed or endorsed by the publisher.
